# Quantitative PSMA-PET parameters in localized prostate cancer: prognostic and potential predictive value

**DOI:** 10.1186/s13014-024-02483-w

**Published:** 2024-07-29

**Authors:** Stephanie Bela Andela, Holger Amthauer, Christian Furth, Julian M. Rogasch, Marcus Beck, Felix Mehrhof, Pirus Ghadjar, Jörg van den Hoff, Tobias Klatte, Rana Tahbaz, Daniel Zips, Frank Hofheinz, Sebastian Zschaeck

**Affiliations:** 1 Corporate Member of Freie Universität Berlin and Humboldt-Universität zu Berlin, Department of Radiation Oncology, Charité – Universitätsmedizin Berlin, Augustenburger Platz 1, 13353 Berlin, Germany; 2 Corporate Member of Freie Universität Berlin and Humboldt-Universität zu Berlin, Department of Nuclear Medicine, Charité – Universitätsmedizin Berlin, Berlin, Germany; 3 Helmholtz-Zentrum Dresden-Rossendorf, PET Center, Institute of Radiopharmaceutical Cancer Research, Dresden, Germany; 4 Corporate Member of Freie Universität Berlin and Humboldt-Universität zu Berlin, Department of Urology, Charité – Universitätsmedizin Berlin, Berlin, Germany; 5 Charité – Universitätsmedizin Berlin, Berlin, Germany; 6 German Cancer Consortium (DKTK), Partner Site Berlin, and German Cancer Research Center (DKFZ), Heidelberg, Germany

**Keywords:** PSMA, Positron emission tomography, Prostate cancer, Prostate-specific membrane antigen, Quantitative PET parameters, Prognostic value

## Abstract

**Background:**

PSMA-PET is increasingly used for staging prostate cancer (PCA) patients. However, it is not clear if quantitative imaging parameters of positron emission tomography (PET) have an impact on disease progression and are thus important for the prognosis of localized PCA.

**Methods:**

This is a monocenter retrospective analysis of 86 consecutive patients with localized intermediate or high-risk PCA and PSMA-PET before treatment The quantitative PET parameters maximum standardized uptake value (SUV_max_), tumor asphericity (ASP), PSMA tumor volume (PSMA-TV), and PSMA total lesion uptake (PSMA-TLU = PSMA-TV × SUV_mean_) were assessed for their prognostic significance in patients with radiotherapy or surgery. Cox regression analyses were performed for biochemical recurrence-free survival, overall survival (OS), local control, and loco-regional control (LRC).

**Results:**

67% of patients had high-risk disease, 51 patients were treated with radiotherapy, and 35 with surgery. Analysis of metric PET parameters in the whole cohort revealed a significant association of PSMA-TV (*p* = 0.003), PSMA-TLU (*p* = 0.004), and ASP (*p* < 0.001) with OS. Upon binarization of PET parameters, several other parameters showed a significant association with clinical outcome. When analyzing high-risk patients according to the primary treatment approach, a previously published cut-off for SUV_max_ (8.6) showed a significant association with LRC in surgically treated (*p* = 0.048), but not in primary irradiated (*p* = 0.34) patients. In addition, PSMA-TLU (*p* = 0.016) seemed to be a very promising biomarker to stratify surgical patients.

**Conclusion:**

Our data confirm one previous publication on the prognostic impact of SUV_max_ in surgically treated patients with high-risk PCA. Our exploratory analysis indicates that PSMA-TLU might be even better suited. The missing association with primary irradiated patients needs prospective validation with a larger sample size to conclude a predictive potential.

*Trial registration* Due to the retrospective nature of this research, no registration was carried out.

**Supplementary Information:**

The online version contains supplementary material available at 10.1186/s13014-024-02483-w.

## Background

Prostate cancer (PCA) accounted for 7.1% of all cancer cases in 2018, being the second most common cancer in men worldwide [1]. Therapeutic options for localized PCA include radical prostatectomy, radiotherapy, and active surveillance. The D’Amico classification is routinely used for clinical risk stratification [2]. While PCA-specific ten-year mortality is negligible for low-risk patients regardless of the chosen therapeutic approach [3], cancer-specific mortality is considerably higher in high-risk patients despite treatment intensification, i.e. concomitant androgen deprivation therapy (ADT), dose-escalated radiotherapy or extended pelvic lymph node dissection [3–6]. Even in intermediate-risk patients around 15% present tumor progression within 5 years after treatment, even with high-dose radiotherapy [7]. Current primary treatment approaches for unfavorable intermediate-risk or high-risk PCA with disease limited to the prostate is either surgery or radiotherapy combined with ADT. While both treatment approaches show a similar oncological outcome, treatment-related side effects differ considerably between both treatment approaches [8]. Additionally, surgical patients usually receive adjuvant or salvage radiotherapy to the prostate fossa in case of biochemical recurrence, leading to a combination of surgical and radiation-induced side effects [9]. Therefore, biomarkers for better treatment personalizations are urgently needed.

Positron emission tomography (PET)/Computer tomography (CT) with prostate-specific membrane antigen (PSMA) is increasingly used for staging high-risk patients due to its high sensitivity and specificity regarding the detection of lymph nodes or distant metastases [10, 11]. The diagnostic accuracy of PSMA-PET/CT for staging therapy-naïve patients [12, 13] and the greater efficacy in detecting early recurrences, small lymph node metastases, and bone metastases [14] has been shown to be superior to bone scintigraphy and CT.

In addition to improved staging of patients, quantitative PET parameters can potentially be used as imaging biomarkers. This holds true for the most frequently investigated parameter SUV_max_ (maximum standardized uptake value, normalized to body weight) which delivers additional prognostic information in various diseases and for various PET tracers [15–17]. Due to the novelty of PSMA-PET imaging, data on the prognostic value of quantitative imaging parameters for this specific tracer is sparse and mostly limited to advanced stages of disease. So far, most data have been published on metastatic patients treated with PSMA radioligand therapy [18, 19]. Two recent publications showed that quantitative PSMA-PET parameters are associated with biochemical recurrence-free survival in PCA patients treated with primary surgery [20, 21].

This study aimed to investigate the prognostic value of various quantitative PSMA-PET parameters, including previously published cutoff values [21], in a cohort of intermediate/high-risk patients. In contrast to existing publications, these analyses were restricted to PET-staged localized PCA treated with curative intent (either surgery or radiotherapy ± ADT), i.e., patients without nodal or distant metastatic disease after PSMA-PET staging.

## Patients and methods

### Patient cohort

For this retrospective analysis, all patients who underwent PSMA-PET at Charité – Universitätsmedizin Berlin, Campus Virchow-Klinikum between January 2015 and December 2018 were reviewed and checked for inclusion and exclusion criteria. Results of imaging and implications for staging of patients included up to March 2018, have been published [22]. For this analysis, we re-evaluated the existing patient cohort and added patients with pre-treatment PSMA-PET until to December 2018, as recently published [23].

This allowed identification of all treatment-naïve patients with biopsy-proven PCA who underwent PSMA-PET/CT for initial staging. Patients were eligible for this analysis if they presented with intermediate/high-risk disease and underwent active therapy, i.e., radiotherapy with/without ADT or surgery, and if at least one follow-up visit was available. Exclusion criteria were patients with detectable lymph nodes or distant metastases by PET imaging. This resulted in a cohort of eighty-six patients. From this cohort, thirty-five patients underwent radical prostatectomy (RP), and fifty-one patients underwent radiotherapy (RT).

Referral for PSMA-PET was at the discretion of the treating urologists and radiation oncologists, particularly because PSMA-PET has not been established for routine staging. The study was approved by the local ethics committee (EA4/168/16).

### Clinical parameters

Clinical data including prostate-specific antigen (PSA), clinical T-stage, and Gleason score assessed at biopsy before imaging were available from electronic databases, as well as from patient records. For the subgroup of patients who underwent surgery, the surgical Gleason scores were recorded. The Gleason scores were classified according to the recommendations of the consensus conference of the International Society of Urological Pathology (ISUP) of 2014 on Gleason grading of PCA [24]. Additionally, ADT usage at the time of PSMA-PET/CT, during active therapy (RT, RP) and during follow-up was assessed.

Based on the clinical parameters, a three-tiered classification was made into low, intermediate, or high-risk patients according to D’Amico scoring. During follow-up, patients usually underwent repeated PSA examinations every three months. In case of biochemical recurrence (BCR), further diagnostic examination was left at the discretion of the treating urologist/radiation oncologist. The endpoints of the study were local tumor control (LC), loco-regional tumor control (LRC), biochemical recurrence-free survival (BRFS), and overall survival (OS). LC and LRC were defined as local/loco-regional recurrence detected by magnetic resonance imaging (MRI) or PSMA-PET. Since surgically treated patients underwent salvage radiotherapy to the prostate fossa usually at low PSA values and therefore did not present macroscopic local recurrence, we used the following approach: A local recurrence after surgery was considered if patients presented a biochemical recurrence with complete biochemical remission after salvage radiotherapy to the prostate fossa. Patients who received additional radiotherapy to the lymphatic drainage had to be excluded in this analysis regarding the endpoint local recurrence as the complete remission after irradiation could also have been due to microscopic regional recurrences. Biochemical recurrence was defined as follows: In patients treated with primary radiotherapy, BCR was defined according to the Radiation Therapy Oncology Group- American Society for Therapeutic Radiology and Oncology (RTOG-ASTRO)-Phoenix consensus recommendation [25] as an increase of 2 ng/ml or more above the lowest PSA level achieved (PSA nadir). After radical prostatectomy, the American Urological Association (AUA) expert panel recommends defining biochemical recurrence as a first serum prostate specific antigen of > or = 0.2 ng/ml, with a second confirmatory PSA value of > 0.2 ng/ml [26].

### Imaging

Imaging was previously described [22], briefly, PSMA-PET/CT was performed with the radiotracer [^68^Ga]Ga-PSMA-HBED-CC on a dedicated PET/CT scanner (Gemini TF 16; Philips, The Netherlands) with Philips Astonish TF technology. Injection of [^68^Ga]Ga-PSMA-HBED-CC was given intravenously (median activity 153 MBq; range: 71–227 MBq), Median activity per kg body weight: 1.6 MBq/kg (range: 0.8–2.6 MBq/kg). PET imaging was conducted at a Median of 98 min after injection (range: 39–188 min). Patients were positioned supine and scanned from the head to the proximal thighs (emission, 90–180 s per bed position; 3D acquisition mode; bed overlap, 53.3%). Attenuation correction was based on non-enhanced low-dose CT (automatic tube current modulation; maximum tube current–time product, 50 mA; tube voltage, 120 kV; gantry rotation time, 0.5 s) reconstructed with a slice thickness of 5 mm (convolution kernel, B08). Raw PET data were reconstructed using iterative reconstruction with TOF analysis (Philips Astonish TF technology; BLOB-OS-TF; iterations, 3; subsets, 33). Projection data were reconstructed with matrix size 144 × 144 and voxel size 4 × 4 × 4 mm^3^ [22].

### Image evaluation

All 3D region of interest (ROI) definitions and image analyses were performed using ROVER software, version 3.0.41 (ABX, Radeberg, Germany). Initially, a large spherical mask was placed around the prostate and the base of the seminal vessels to delineate the metabolically active part of the primary tumor based on a threshold of 41% SUV_max_, as suggested in a recent analysis [20].

The resulting ROIs were visually analyzed by an experienced observer (SZ) to manually exclude tracer uptake of surrounding normal tissue (bladder and/or rectum). An example of delineation is shown in Supplementary Fig. 1. In patients with low diffuse tracer accumulation in the prostate, the most intense single voxel was manually delineated for calculation of Maximum standardized uptake value (SUV_max_). SUV_max_, average standardized uptake value (SUV_mean_), the delineated tumor volume according to PSMA uptake (PSMA-TV) and the PSMA total lesion uptake (PSMA-TLU = PSMA-TV × SUV_mean_) were calculated. In addition, the novel quantitative PET parameter tumor asphericity (ASP) was calculated. ASP calculation was described in detail before. ASP is defined as

$${\text{ASP}} = 100*\left( {\sqrt[3]{H} - 1} \right)\quad {\text{with}}\quad H = \frac{{1\;S^{3} }}{{36\pi V^{2} }}$$

where S und V are surface and volume of the lesion and it measures the fractional increase of the surface area of a lesion compared to the surface area of spherical lesion with the same volume (which would have a value of zero). More details can be found in [23, 27, 28].

### Statistical analyses

Differences between groups (surgery vs. radiotherapy) were analyzed by Mann–Whitney U test. The association of BRFS, OS, LRC and LC, measured from start of therapy to death and/or event, with clinically relevant parameters as well as quantitative PET parameters was analyzed using univariable Cox proportional hazards regression in which the PET parameters were included as metric parameters. In a second step, the PET parameters were binarized and again analyzed in univariable Cox regression. For SUV_max_ the previously published value of 8.6 was used for this purpose. The cutoff values for all other parameters were calculated by performing a univariable Cox regression for each measured value. In each variable, the value leading to the hazard ratio (HR) with the highest significance was used as cutoff. Cutoff values for the clinical parameters age and iPSA were computed accordingly. The cutoff values were separately computed for each endpoint. The probability of survival was computed and rendered as Kaplan–Meier curves. Statistical significance was assumed at a *p* value of 0.05 or lower. Statistical analysis was performed with the *R language and environment for statistical computing* version 4.2.3 [29].

## Results

### Patient cohort

**Fig. 1:**
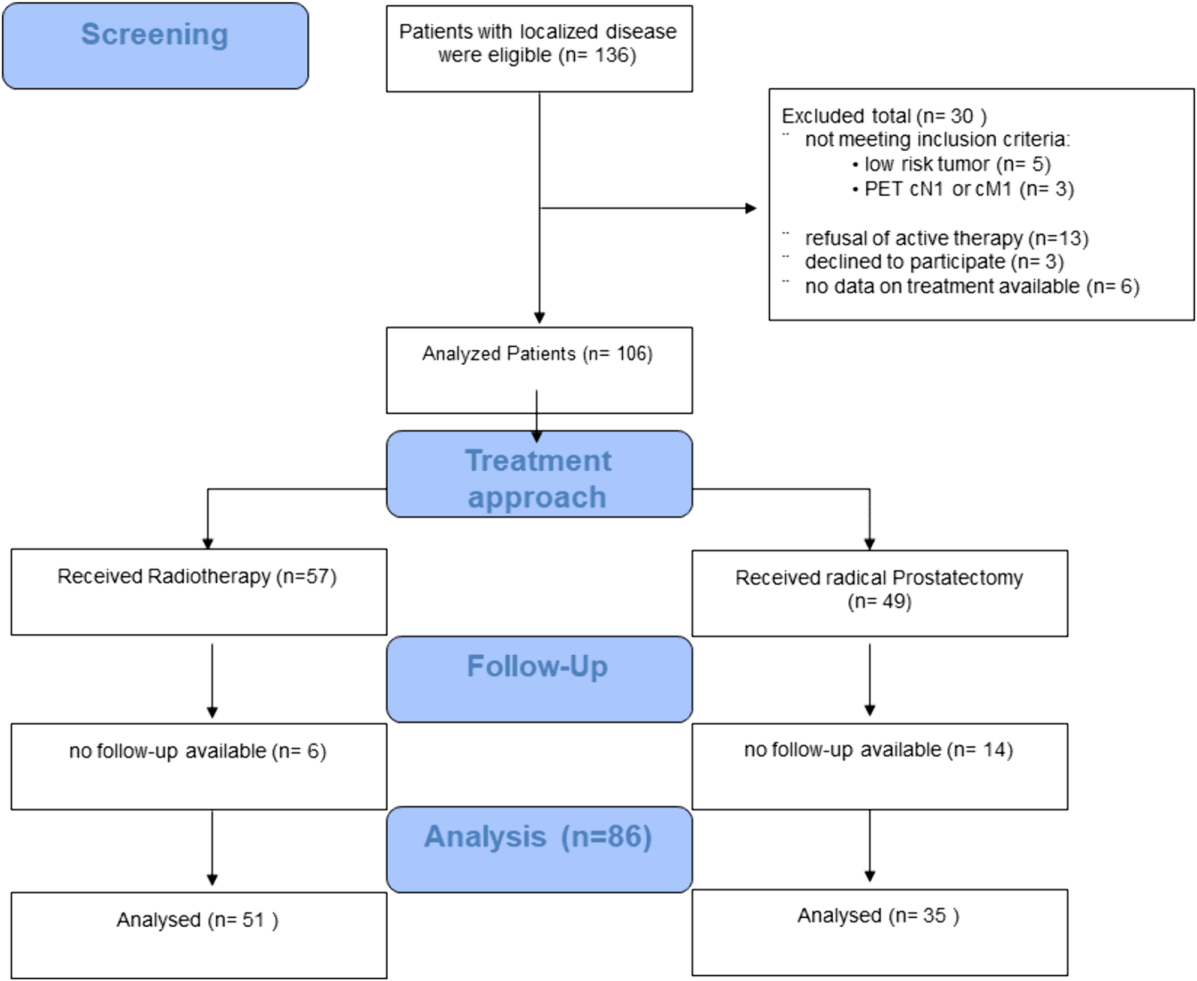
Flow Diagram of all patients with PSMA-PET imaging for primary prostate cancer during the study period and patients that have been analyzed

During the study period, 136 patients underwent PSMA-PET/CT staging for treatment naïve localized PCA. After excluding all patients that did not meet the specified inclusion criteria, eighty-six patients were evaluable for further analyses. Exclusion was most frequently due to low-risk tumors, refusal of any therapy by the patient or missing data on treatment or follow-up information. Figure 1 shows a flowchart of all patients that have been screened for this study. Median follow up time in analyzed patients was 42 months. In the whole cohort thirteen patients had a biochemical relapse and seven patients died during follow-up. In the intermediate risk biochemical relapse/death occurred in three/one patients, in the high-risk group there were ten/six event.

Table 1 summarizes the patient and clinical tumor characteristics for all patients (D’Amico intermediate and high risk groups).

**Table 1:** Patient characteristics of all Patients (D’Amico 2 and 3, n = 86)

Mean age (range)	75.5 (61–87)
Mean PSA (range)	11.6 (2.55–130.5)
Gleason Score (biopsy)	
≤ 6	9 (10.47%)
7a	9 (10.47%)
7b	17 (19.77%)
8	36 (41.86%)
9	14 (16.28%)
10	1 (1.16%)
Clinical T stage	
n/a	16 (18.60%)
1	42 (48.84%)
2	19 (22.09%)
3	8 (9.30%)
4	1 (1.16%)
D’Amico risk group	
Intermediate risk	27 (31.40%)
High risk	59 (68.60%)
Gleason Score (surgery)	
n/a	52 (60.47%)
≤ 6	0 (0%)
7a	7 (8.14%)
7b	16 (18.60%)
8	2 (2.33%)
9	9 (10.47%)
10	0 (0%)

### Association of quantitative PET parameters with clinical outcome in the whole cohort

Analysis of metric PET parameters revealed a significant association of PSMA-TV (*p* = 0.003), PSMA-TLU (*p* = 0.004) and ASP (*p* < 0.001) with OS as shown in Table 2.

**Table 2:** Univariable Cox regression for all patients (intermediate and high-risk)

Parameter	HR	95% CI	*p* value	HR	95% CI	*p* value
	*BCR*			*OS*		
Age > 74 y	0.29	0.09–1	**0**.**049**	5.97	1–35.75	0.051
iPSA > 11	1.57	0.67–3.69	0.3	3.87	0.64–23.28	0.14
Gleason score > 8	1.35	0.5–3.68	0.55	2.7	0.3–24.17	0.37
D Amico Score > 2	1.46	0.53–3.97	0.46	1.38	0.15–12.41	0.77
PSMA-TV	0.96	0.89–1.04	0.31	1.16	1.05–1.28	**0**.**003**
PSMA-TLU	0.998	0.992–1.004	0.45	1.005	1.002–1.009	**0**.**004**
SUVmax	1	0.98–1.019	0.97	0.97	0.89–1.06	0.48
ASP	0.99	0.96–1.02	0.52	1.05	1.02–1.07	< **0**.**001**
	*LRC*			*LC*		
Age > 74 y	0.36	0.08–1.6	0.18	3.23	0.4–26.41	0.27
iPSA > 11	3.62	0.99–13.17	0.051	7.58	0.93–61.67	0.058
Gleason score > 8	1.62	0.5–5.28	0.42	1.62	0.32–8.04	0.56
D Amico Score > 2	1.36	0.37–5.02	0.65	1.39	0.28–6.96	0.69
PSMA-TV	0.98	0.9–1.07	0.64	0.98	0.88–1.09	0.73
PSMA-TLU	0.999	0.993–1.005	0.75	0.993	0.977–1.009	0.39
SUVmax	1.004	0.981–1.028	0.72	0.97	0.91–1.04	0.41
ASP	0.995	0.959–1.032	0.79	0.991	0.942–1.043	0.73

PET parameters were included as metric parameters. Bold *p* values indicate significance (*p* < 0.05)

Upon binarization of PET parameters, ASP, PSMA-TV and PSMA-TLU showed a significant association or a statistical trend for OS. In addition, an association with several other investigated endpoints was observed, as shown in Table 3.

**Table 3:** Univariable Cox regression for all patients (intermediate and high-risk)

Parameter	Risk	HR	95% CI	*p* value
*BCR*				
PSMA-TV	> 7.8 ml	0.17	0.02–1.26	0.083
PSMA-TLU	> 33.6 ml	0.48	0.19–1.17	0.1
SUVmax	> 8.6	1.63	0.64–4.17	0.31
ASP	> 7.22%	0.42	0.18–0.98	**0**.**046**
*OS*				
PSMA-TV	> 14.9 ml	34	3.7–312.7	**0**.**002**
PSMA-TLU	> 76 ml	9.58	1.07–85.74	**0**.**043**
SUVmax	> 8.6	1.91	0.21–17.14	0.56
ASP	> 31.9%	19.9	2.22–178.41	**0**.**0075**
*LRC*				
PSMA-TV	> 1.48 ml	4.84	0.62–37.58	0.13
PSMA-TLU	> 112 ml	2.85	0.87–9.29	0.083
SUVmax	> 8.6	3.26	0.72–14.74	0.12
ASP	> 7.22%	0.45	0.15–1.34	0.15
*LC*				
PSMA-TV	> 4 ml	0.21	0.03–1.7	0.14
PSMA-TLU	> 30.3 ml	0.13	0.02–1.04	0.054
SUVmax	> 8.6	1.66	0.33–8.22	0.54
ASP	> 6.89%	0.29	0.07–1.2	0.088

PET parameters were included as binarized parameters

### Quantitative PET parameters in high-risk patients

Since most data are available for high-risk patients and this group is of greatest interest for treatment personalization, high-risk patients were evaluated seperately. Results for binarized PET parameters were similar. Details are shown in Supplementary Table 1. Supplementary Table 2 summarizes the patient and clinical tumor characteristics for the high-risk patients according to the primary treatment approach, radiotherapy vs. radical prostatectomy, and shows results of statistical group comparison. Fifty-nine men met high-risk criteria, of whom 26 underwent RP and 33 received RT. Baseline characteristics were similar in the two groups except for age. Patients in the radiotherapy group were significantly older (*p* = 0.04).

### Quantitative PET parameters in patients according to primary treatment approach

**Fig. 2:**
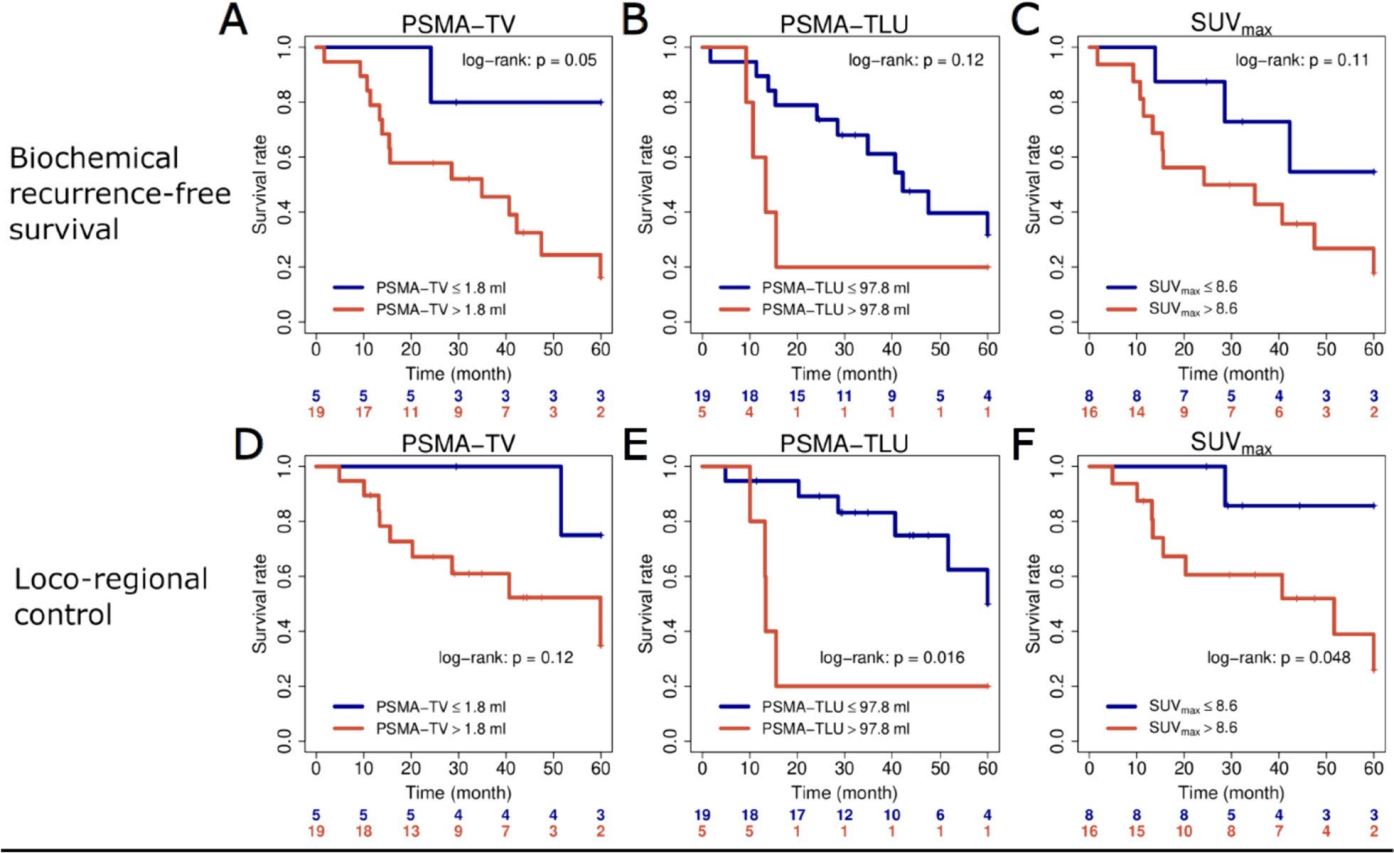
Quantitative PET parameters PSMA-TV (**A**, **D**), PSMA-TLU (**B**, **E**) and SUV_max_ (**C**, **F**) in surgically treated high-risk patients. Kaplan–Meier plots for the investigated endpoints biochemical recurrence-free survival (**A**–**C**): and loco-regional control (**D**–**F**)

Finally, all quantitative PET parameters were analyzed according to primary treatment approach. While cut-offs for PSMA-TV and PSMA-TLU were optimized for each clinical endpoint, for SUV_max_ a previously published cut-off was chosen for each endpoint. Figure 2 shows results for these three PET parameters in surgically treated high-risk patients.

**Fig. 3:**
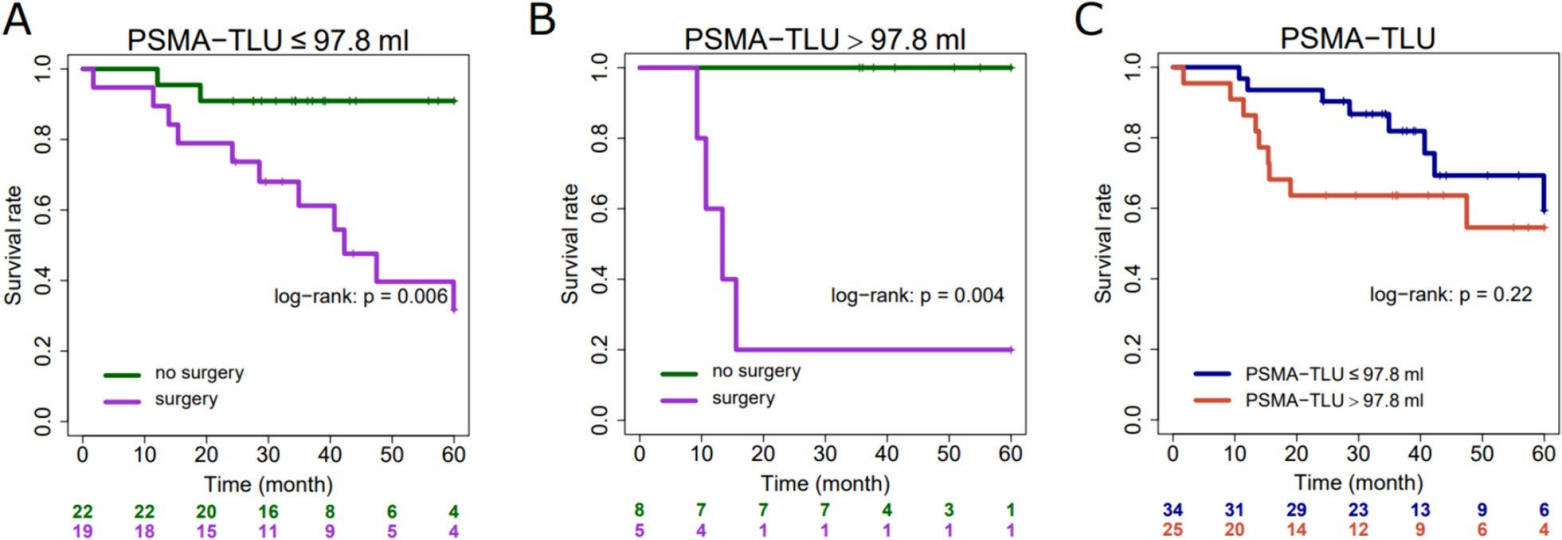
Risk for biochemical recurrence according to primary treatment approach (surgery vs. no surgery, i.e., radiotherapy or ADT plus radiotherapy). Patients are stratified according to TLU 97.8 and treatment approach: Patients with TLU below 97.8 (**A**) and above 97.8 (**B**). Outcome of the whole cohort is shown on **C**

PSMA-TV significantly stratified patients at high versus low-risk for biochemical recurrence (*p* = 0.05), and PSMA-TLU showed a significant discrimination for patients at high versus low-risk for loco-regional tumor recurrence (*p* = 0.016). The previously published cut-off value SUV_max_ = 8.6 which was established in surgically treated patients, showed a significant discrimination between patients at high versus low risk for loco-regional recurrence after surgical treatment (*p* = 0.048). The same analyses in patients treated with primary radiotherapy did not reveal a significant association with outcome, including SUV_max_ = 8.6 with the endpoint LRC (*p* = 0.34), there were also no significant associations of quantitative PET metrics with BCR (see Supplementary Fig. 2). Results for surgical patients remained comparable when including intermediate and high-risk patients, as shown in Supplementary Fig. 3. As PSMA-TLU 97.8 showed the highest potential for risk stratification in surgical patients, this parameter was further analyzed. PSMA-TLU > 97.8 does not seem to be associated with risk for biochemical recurrence in irradiated patients as irradiated patients with higher values presented no case of biochemical recurrence as shown in Fig. 3.

## Discussion

Here, we report the most comprehensive analysis of quantitative PSMA PET parameters and patient outcome for localized PCA treated with curative intent. To our best knowledge, this is the first evaluation of patients treated with primary radiotherapy, as two recent publications only evaluated surgically treated patients [20, 30]. While it was possible to validate a previously published cut-off for SUV_max_ in surgically treated patients [21, 30], it was not possible to validate this cutoff in patients treated with primary radiotherapy.

Recent ex vivo analyses for radiosensitivity seem to encourage the potential of PSMA-PET as a predictive biomarker for radiotherapy. Marinescu and colleagues used an assay with residual γH2AX foci after ex vivo radiotherapy and reported a positive correlation between radiation-induced γH2AX foci, i.e. a surrogate of irradiation-induced DNA double-strand breaks, and PSMA uptake, measured by SUV_max_ [31]. These analyses would suggest an association between radiosensitivity and higher uptake of PSMA.

Our results suggest that quantitative PET parameters have prognostic potential in terms of disease progression and outcome in high-risk patients with surgically treated PCA, which may allow better stratification of patients. This could mean that these patients should preferentially be treated by primary radiotherapy and ADT or, if surgery has to be performed, these high-risk patients could for example receive neoadjuvant ADT before surgery with the aim to reduce the risk for local recurrence by downsizing. While it was possible to validate a recently published cut-off value for SUV_max_ [21], other quantitative parameters have not been investigated in this setting before. Our preliminary analyses suggest that PSMA-TV and PSMA-TLU might be even better quantitative parameters regarding risk stratification. The missing association of the quantitative parameters with primary irradiated patients could indicate a potential predictive value that might be used for treatment guidance in adjunct to other key factors, i.e. side effects of the chosen therapeutic approach and individual patient preference.

Several recent studies investigated the prognostic value of quantitative PSMA-PET parameters. Most of these studies analyzed advanced stage disease. For example, PSMA uptake has been associated with prognosis and outcome in advanced metastatic disease. Calderoni and colleagues reported an association between SUV_max_ and treatment response in 160 metastatic castration-resistant PCA patients undergoing various systemic treatments [32]. Due to extensive PET imaging, most data is available for patients undergoing PSMA radioligand therapy. In a small cohort of 40 patients with advanced PCA and radioligand therapy, Seifert and colleagues reported that semiautomatically quantified pre-treatment tumor volume (PSMATV50) was significantly associated with OS and independent from other important prognostic factors like alkaline phosphatase (ALP) and PSA [33]. In another study with 38 patients who underwent [^177^Lu]Lu-PSMA-617 radioligand therapy (RLT), the change in total tumor volume before and after RLT was significantly associated with PSA response and overall survival after a median follow-up of 17 months [19]. A recent retrospective multicenter analysis of 237 men treated with RLT established the tumor-to-salivary gland ratio as a prognostic factor, as the authors were able to show an association with PSA response and OS [34].

Given the available data in metastatic disease, determining quantitative imaging parameters as an indicator of disease progression is also of interest in less advanced tumor stages. So far, only few studies investigated patients with localized PCA. It is important to note that in these stages OS is usually not reported due to the short follow-up period and often particularly good prognosis in low- and intermediate-risk patients. Therefore, surrogate parameters are frequently used, for example, biochemical recurrence, measured by serum PSA. However, these surrogate markers are controversial since the association with important clinical outcome parameters like OS is only weak [35, 36]. This limitation also holds true for our study, as there were only few deaths during the follow-up period. Additionally, it is not clear if these events were caused by tumor aggressiveness or due to comorbidities in an old patient population. Regarding BCR, its definition is subject to different criteria and depends on the type of local therapy: Prostatectomy or radiotherapy [25, 26]. Differences in the definition of PSA relapse thus have an additional impact on comparability. Due to the known limitations of BCR [37, 38], we included local control and loco-regional control as additional, clinical more relevant endpoints. So far only data on the association of quantitative PSMA PET parameters and BCR after surgery has been published.

Xuefeng et al. reported good efficacy in predicting biochemical recurrence (BCR) using PET-CT before prostatectomy. They found a significant association between SUV_max_ and BCR (*p* < 0.01) [39]. Another large retrospective cohort study with 848 men also supports the association between quantitative PSMA parameters and patient outcome: Roberts and colleagues reported that PSMA intensity measured by SUV_max_ is a novel independent prognostic factor for biochemical recurrence-free survival (BRFS) after radical prostatectomy. PSMA intensity was significantly associated with a shorter time to biochemical recurrence [30]. This corroborates previous reports by Wang and colleagues who found an association of SUV_max_ and SUV_mean_ with BCR in 186 surgically treated patients [40]. Interestingly, this is one of the few studies, that investigated other quantitative PET parameters like tumor volume and total lesion uptake. Contrary to our findings, these other parameters did not perform better than SUV, which might indicate an accidental finding due to the small numbers of patients in our study. In another study, SUV_max_ of the primary tumor was associated with pre-and postoperative variables as it was highly associated with known conventional prognostic factors such as the International Society of Urological Pathology (ISUP) pathological score and lymph node status [41].

In our study, we successfully confirmed previous evidence on the prognostic value of quantitative PET parameters in surgically treated patients with high-risk PCA by applying a published cut-off for SUV_max_ [21, 30]. Our study has several strengths: it includes extensive imaging evaluation of different quantitative PET parameters and is restricted to patients without evidence of metastases by all imaging techniques, including PSMA-PET. We think the latter is particularly important because otherwise the prognostic accuracy of PET parameters might be heavily confounded by the better diagnostic accuracy to detect lymph nodes or distant metastases (both factors have a known strong detrimental effect on patient outcome [20]). In addition, the same PSMA tracer and PET device were used in all patients, facilitating comparability of PET parameters between individual patients. Nevertheless, our data must be interpreted as preliminary and hypothesis-generating because our study also has several important limitations. First, this was a retrospective evaluation with a population of a single center. Second, the quantitative values were collected retrospectively and without standardization (e.g., in terms of recording time), so they may be subject to measurement and reader variation but also reflect a real-world application environment. A major limitation is the high variability of injected dose per kg. We think this can partially be explained by the long follow-up of analyzed patients. One must bear in mind that these patients have been scanned immediately after the introduction of PSMA-PET. Therefore, standardized protocols were not available at that time for this specific tracer. As an additional limitation, the PET scanner is therefore not the most recent one and has a lower resolution compared to the newest generation scanners. In modern PET scanners, voxel sizes are usually smaller than in the scanner that was used for the current analysis, owing to the improved intrinsic spatial resolution and/or system sensitivity of these scanners. In small target lesions, a larger voxel size could result in an underestimation of the SUV_max_ compared to a scanner or reconstruction with a smaller voxel size. This effect would have to be considered when attempting to harmonize SUV values, for example, through the use of adaptive Gaussian filters [42].

Especially the high variability in injected dose and scan duration directly affects the SUV quantification. Additionally, it is a well-known phenomenon that the comparability of SUVs across different PET scanners or reconstruction methods is limited. The scanner used for the current analysis complies with the initial standard set by the EANM Research GmbH’s (EARL) initiative in 2010. It has already been demonstrated that SUV values from newer generation scanners, which adhere to the updated EARL standard [43], cannot be directly compared, and established prognostic SUV thresholds may not necessarily be transferrable [44]. Consequently, when transferring the currently used thresholds or quantitative (SUV) results to new PET scanners, correction methods or harmonization steps would have to be applied [42]. This is particularly true for small lesions, whose activity or SUV might be underestimated due to the limited reconstructed spatial resolution of the used scanner compared to newer scanners or advanced reconstruction methods.

However, it should be noted that even in this suboptimal setting we were able to confirm the previously published prognostic value of SUV, underlining the validity of these results. Third, the follow-up time of patients is still relatively short and needs to be confirmed by longer follow-up. As mentioned earlier, this is particularly true for OS, which would require 10 years of follow-up even in high-risk patients. Another limitation regards the relatively small number of patients, especially when analyzing sub-groups. Due to the small sub-groups, we did not perform multivariate analyses, which is another limitation of the current study. Due to the small number of patients, our results should be interpreted as hypothesis generating. Especially the association of quantitative PET parameters with outcome in surgically treated, but not in irradiated patients. While these findings are of great interest since they could indicate a predictive biomarker that could potentially be used for better treatment decisions, these findings must be regarded as exploratory. It is an important limitation that the number of events in the group of irradiated patients is low, therefore the missing statistical association might only be due to this. In addition, our chosen approach to classify complete biochemical response after salvage radiotherapy as local recurrence might be debatable, as local control is usually defined based on follow-up imaging. Furthermore, clinical and pathological staging might differ in surgical patients, as previously reported [23]. We decided to compare patients according to clinical staging, as this would be the information available for treatment decisions in a clinical setting.

## Conclusion

Our exploratory analysis suggests that SUV_max_, PSMA-TV and PSMA-TLU are promising parameters for better patient stratification in curatively treated high and intermediate-risk PCA patients. Quantitative PSMA-PET parameters might also have a predictive value regarding the chosen therapeutic approach. After successful prospective validation of these findings, these parameters could aid physicians in treatment selection and shared decision making.

### Supplementary Information

Supplementary Material 1.

## Data Availability

The datasets used and/or analysed during the current study are available from the corresponding author on reasonable request.

## Abbreviations

ADT: Androgen deprivation therapy
ALP: Alkaline phosphatase
ASP: Tumour asphericity
AUA: American Urological Association
BRFS: Biochemical recurrence-free survival
CT: Computer tomography
ISUP: International Society of Urological Pathology
LC: Local control
LRC: Loco-regional control
MRI: Magnetic resonance imaging
OS: Overall survival
PCA: Prostate cancer
PET: Positron emission tomography
PSA: Prostate specific antigen
PSMA-PET: Prostata-Specific Membrane Antigen-positron emission tomography
PSMA-TLU: PSMA total lesion uptake
PSMA-TV: PSMA tumor volume
PSMATV50: Pre-treatment tumor volume
RLT: Radioligand therapy
ROI: Region of interest
RTOG-ASTRO-Phoenix: Radiation Therapy Oncology Group-American Society for Therapeutic Radiology and Oncology Phoenix
RP: Radical prostatectomy
RT: Radiotherapy
SUV_max_: Maximum standardized uptake value
SUV_mean_: Mean standardized uptake value
SZ: Sebastian Zschaeck
