# Supplementary material for: Quantitative PSMA-PET parameters in localized prostate cancer: prognostic and potential predictive value
Source: Radiat Oncol. 2024 Jul 29;19:97. doi: 10.1186/s13014-024-02483-w (PMC11288109; doi:10.1186/s13014-024-02483-w)

**Quantitative PSMA-PET parameters in localized prostate cancer: prognostic and potential predictive value**

**Supplementary material**

**Supplementary table 1:** Univariate Cox regression for high risk patients. PET parameters were included as binarized parameters.

| Parameter | Risk | HR | 95% CI | P value |
| --- | --- | --- | --- | --- |
| **BCR** |  |  |  |  |
| PSMA-TV | *>* 2.03ml | 6.5 | 0.86 – 49.1 | **0.07** |
| PSMA-TLU | *>* 14.2ml | 4.29 | 0.57 – 32.39 | 0.16 |
| SUVmax | *>* 8.6 | 2.37 | 0.68 – 8.25 | 0.18 |
| ASP | *>* 7.13% | 0.49 | 0.19 – 1.28 | 0.14 |
| **OS** |  |  |  |  |
| PSMA-TV | *>* 17.2ml | 31.35 | 2.95 – 332.6 | **0.004** |
| PSMA-TLU | *>* 137ml | 5.73 | 0.8 – 40.97 | 0.082 |
| SUVmax | *>* 8.6 | 1.18 | 0.12 – 11.41 | 0.89 |
| ASP | *>* 33.7% | 18.44 | 1.91 – 177.75 | **0.012** |
| **LRC** |  |  |  |  |
| PSMA-TV | *>* 2.03ml | 3.93 | 0.5 – 31.15 | 0.2 |
| PSMA-TLU | *>* 112ml | 3.24 | 0.9 – 11.62 | 0.071 |
| SUVmax | *>* 8.6 | 4.72 | 0.6 – 37.34 | 0.14 |
| ASP | *>* 28.8% | 2.23 | 0.57 – 8.7 | 0.25 |
| **LC** |  |  |  |  |
| PSMA-TV | *>* 4ml | 0.24 | 0.03 – 2.07 | 0.19 |
| PSMA-TLU | *>* 30.3ml | 0.14 | 0.02 – 1.23 | 0.077 |
| SUVmax | *>* 8.6 | 2.51 | 0.29 – 21.59 | 0.4 |
| ASP | *>* 31.9% | 3.01 | 0.55 – 16.45 | 0.2 |

**Supplementary Table 2:** Patient characteristics of high-risk Patients Radiotherapy vs radical prostatectomy (D’Amico 3, n= 59).

| **Treatment approach** | All high-risk | RT (n=33) | OP (n=26) | | Mann-Whitney U test |
| --- | --- | --- | --- | --- | --- |
| **Median age**  (range) | 73  (55 – 83) | 73  (61-83) | 70  (55-79) | p = 0.04 | |
| **Median PSA**  (range) | 12,4  (2,55– 130,5) | 16,43  (3,37-130,5) | 11,6 (2,55-50) | P = 0.073 | |
| **Gleason Score** (biopsy) |  |  |  |  | |
| n/a | 0 (0%) | 0 (0%) | 0 (0%) | P = 0.14 | |
| ≤ 6 | 4 (6.78%) | 3 (9.09%) | 1 (3.85%) |  | |
| 7a | 1 (1.69%) | 1 (3.03%) | 0 (0%) |  | |
| 7b | 4 (6.78%) | 2 (6.06%) | 2 (7.69%) |  | |
| 8 | 35 (59.32%) | 21 (63.64%) | 14 (53.85%) |  | |
| 9 | 14 (23.73%) | 6 (18.18%) | 8 (30.77%) |  | |
| 10 | 1 (1.69%) | 0 (0%) | 1 (3.85%) |  | |
| **Clinical T stage** |  |  |  |  | |
| n/a | 8 (13.56%) | 7 (21.21%) | 1 (3.85%) |  | |
| 1 | 27 (45.76%) | 13 (39.39%) | 14 (53.85%) | P = 0.34 | |
| 2 | 17 (28.81%) | 7 (21.21%) | 10 (38.46%) |  | |
| 3 | 6 (10.17%) | 5 (15.15%) | 1 (3.85%) |  | |
| 4 | 1 (1.69%) | 1 (3.03%) | 0 (0%) |  | |

**Supplementary figure 1**: Example of PSMA-PET delineation of one prostate tumor.

**
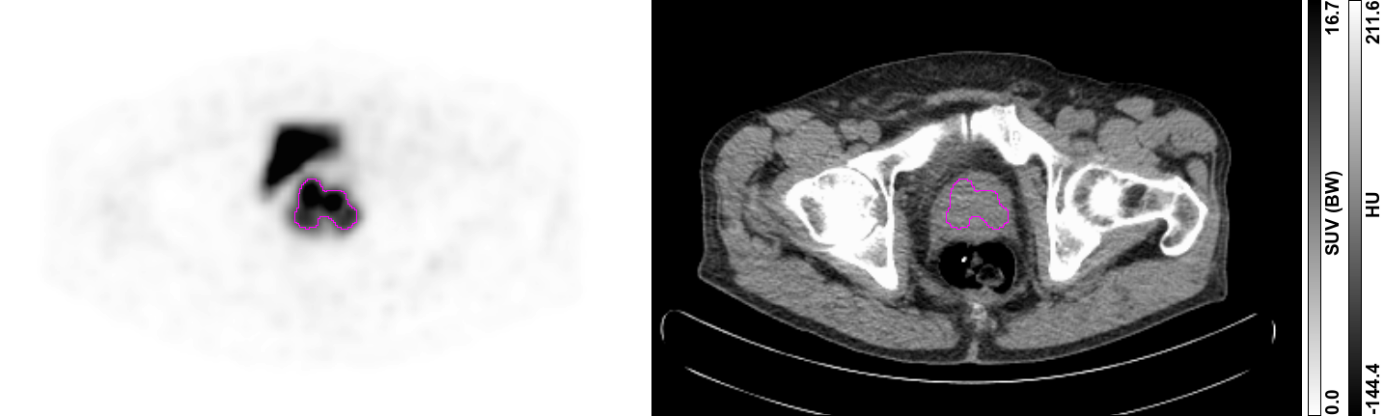
**

**Supplementary figure 2**: Quantitative PSMA-PET metrics in high-risk patients treated with primary radiotherapy.


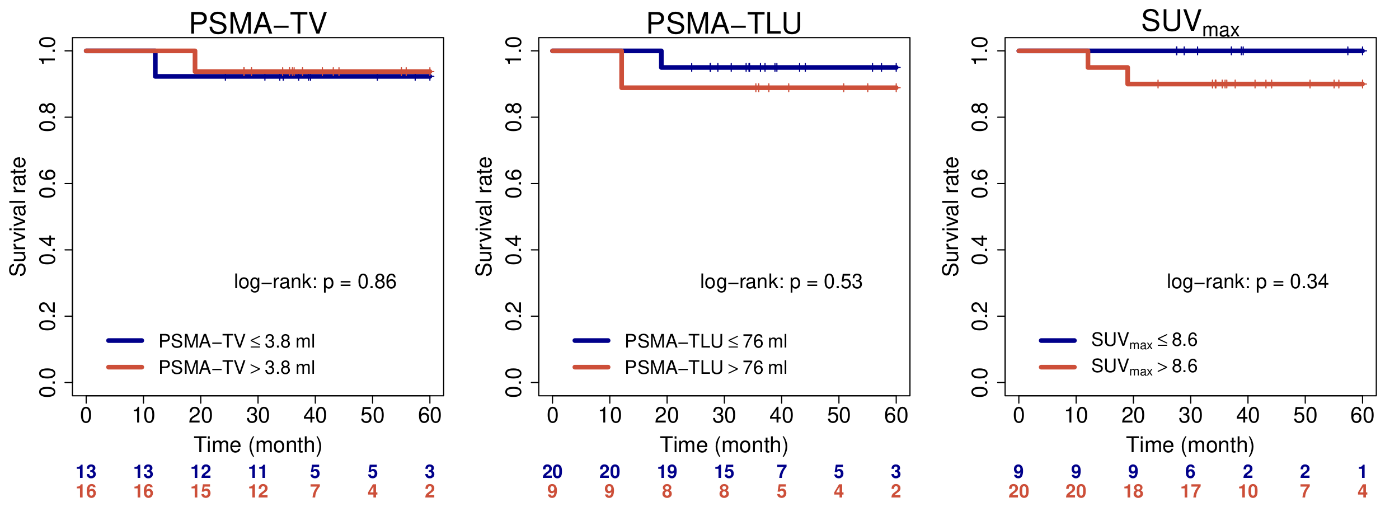


**Supplementary figure 3**: Quantitative PSMA-PET metrics in intermediate and high-risk patients treated with primary surgery.


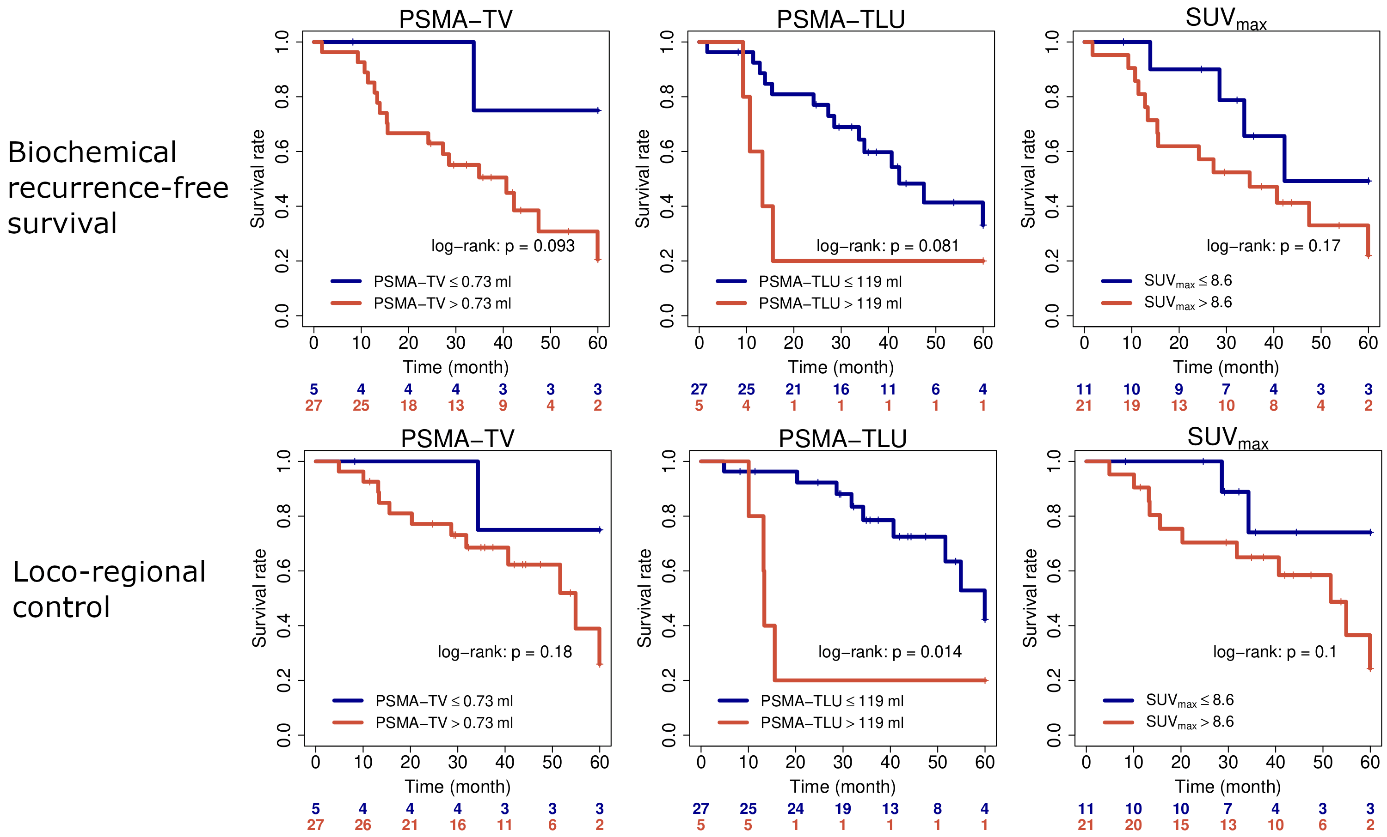

Supplement: Supplementary file 1 — Supplementary Material 1. [file 13014_2024_2483_MOESM1_ESM.docx]
